# Probing the Surface Chemistry of Lithium Nitridation

**DOI:** 10.1021/jacs.5c11781

**Published:** 2025-10-27

**Authors:** Ane Etxebarria, Pinar Aydogan Gokturk, Yifan Ye, Philip N. Ross, Ethan J. Crumlin, Miguel Ángel Muñoz-Márquez

**Affiliations:** † Centre for Cooperative Research on Alternative Energies (CIC energiGUNE), Basque Research and Technology Alliance (BRTA), Alava Technology Park, Albert Einstein 48, Vitoria-Gasteiz 01510, Spain; ‡ Departamento de Física de la Materia Condensada, Facultad de Ciencia y Tecnología, Universidad del País Vasco, UPV/EHU, P.O. Box 644, Bilbao 48080, Spain; § Advanced Light Source, Lawrence Berkeley National Laboratory, Berkeley, California 94720, United States; ∥ Department of Chemistry, Koc University, Istanbul 34450, Turkiye; ⊥ Joint Center for Artificial Photosynthesis, Lawrence Berkeley National Laboratory, Berkeley, California 94720, United States; # Chemical Sciences Division, Lawrence Berkeley National Laboratory, Berkeley, California 94720, United States; ∇ Materials Sciences Division, Lawrence Berkeley National Laboratory, Berkeley, California 94720, United States; ○ School of Science and Technology, Chemistry Division, University of Camerino, via Madonna delle Carceri- ChIP, Camerino, Macerata 62032, Italy

## Abstract

Chemical synthesis of Li_3_N through lithium nitridation has potential to advance rechargeable battery and nitrogen fixation technology. However, studies of the conditions for forming Li_3_N on the lithium surface via nitrogen gas exposure report contradictory findings, such as the spontaneous reaction of Li with pure N_2_, the impossibility of forming Li_3_N through pure Li and N_2_ interaction, the requirement of trace H_2_O to catalyze the reaction, and evidence to the contrary. In this study, ambient pressure X-ray photoelectron spectroscopy (APXPS) was applied to evaluate the in situ chemical evolution of the lithium metal surface under nitrogen gas up to 800 mTorr. At pressures ≤10 mTorr, no Li_3_N was detected. At higher pressures, surface Li_3_N rapidly reacts with trace CO_2_. Additionally, because metallic lithium is readily oxidized by trace gases, the atomic nitrogen concentration of the lithium surface remains below 2%. When nitridation follows oxidation by O_2_ gas, CO_2_ gas, or H_2_O vapor, surface Li_3_N formation is inhibited. These results suggest that nitrogen gas can diffuse through the oxidized lithium metal surface to react with subsurface metallic lithium.

## Graphical abstract

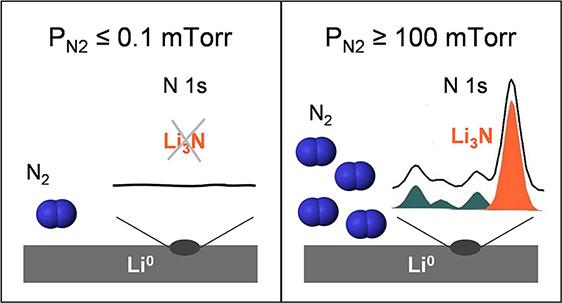

## Introduction

The surface nitridation of metallic lithium is a notable reaction that could advance critical technology, such as lithium metal-based rechargeable batteries, nitrogen fixation to produce valuable chemicals, and emerging Li–N_2_ battery technology, which integrates both energy storage and nitrogen fixation.^1–3^ For Li metal-based batteries, Li_3_N has been proposed as a stabilization layer to protect the lithium metal anode from degradation during electrochemical cycling.^4–8^ Synthesis of Li_3_N in lithium anodes has been conducted through the direct reaction between metallic lithium and nitrogen gas.^5,7,8^ However, due to the highly reactive nature of metallic lithium and the presence of a triple bond in the N_2_ molecule, the role of the purity of both lithium and nitrogen gas in determining the reaction’s outcome has been widely debated. Contradictory results have been reported in the literature since the discovery of Li_3_N by Deslandres in 1895.^9^ For instance, in that same year, Güntz reported that lithium nitride formation was achieved only upon metal exposure to wet nitrogen.^10^ Fifteen years later, in contrast to these first observations, nonreproducible formation of lithium nitride was reported after metal exposure to dry nitrogen at room temperature.^11^

In the second half of the 20th century, lithium nitridation was still of great interest to the scientific community. Kinetic studies were published by McFarlane et al. for solid and molten lithium and dry N_2_.^12^ Shortly thereafter, the role of moisture in the nitridation reaction was further studied. It was shown that pure metal is resistant to nitridation, while the presence of LiOH accelerates Li_3_N formation.^13^ In 1978, a review on lithium’s properties and interactions summarized that lithium nitridation is only possible under two conditions: in the presence of moist nitrogen or under dry conditions at temperatures above 160 °C.^14^ More recently, studies have shown the growth of a Li_3_N overlayer on lithium foil through direct interaction with nitrogen flow at room temperature.^5,7^ However, surface-sensitive spectroscopic analysis revealed significant amounts of contaminants on those surfaces, which were attributed to exposure of the lithium to trace humidity and air. In 2017, Li et al. studied the specific role of water vapor in lithium nitridation. They observed that dry N_2_ results in the formation of a uniform Li_3_N passivation layer on lithium, whereas nitrogen gas with trace water vapor leads to the formation of a porous layer of LiOH and Li_3_N.^15^ Additionally, Ishiyama et al. could not detect Li–N when using H_2_O/N_2_ mixing gas for nitridation.^16^ Spontaneous formation of Li_3_N on lithium at ambient conditions has also been corroborated by theoretical studies.^17^ Other studies have shown that Li_3_N is not formed solely by the interaction of the gas on highly pure metal surfaces.^18,19^

To determine the surface composition and gain clarity on the role of trace gases in lithium nitridation, we employed ambient pressure X-ray photoelectron spectroscopy (APXPS) to directly track chemical changes on the lithium surface during the reaction. We studied the nitridation of lithium on metallic surfaces using gases with 99.999% purity, at pressures up to 800 mTorr, and at room temperature. We found that the formation of Li_3_N depends on the gas pressure and that the selectivity of the lithium–N_2_ gas reaction is much lower than that of lithium reacting with O_2_ and H_2_O vapor, even at concentrations approximately five orders lower than the N_2_ gas pressure. Additionally, we observed that surface Li_3_N is highly reactive: it acts as a precursor to form products that appear consistent with carbon nitrides at room temperature. It was observed that the presence of LiOH on the metallic lithium surface does not promote the growth of Li_3_N. These findings reflect the complexity of creating a pure Li_3_N layer on Li metal by N_2_ gas interaction.

## Experimental Methods

The experiments were carried out at Beamline 9.3.2 of the Advanced Light Source, Lawrence Berkeley National Laboratory. This beamline is equipped with an ambient pressure X-ray photoelectron spectroscopy (APXPS) instrument, which has a Scienta R400 HiPP analyzer and works in the soft X-ray range (between 200 and 900 eV). XPS spectra can be collected with gas pressures up to 1.5 Torr. Prior to dosing gases, the main chamber base pressure was in the low 10^–9^ Torr. The incident photon angle of the instrument is 15°. A quadrupole mass spectrometer (QMS), residual gas analyzer (RDA), AMATEK Dycor was used to monitor the gas dosing. It was connected to the second differential pumping stage, between the reaction chamber and the analyzer, and no electron multiplier was used during the measurements.

The lithium used here was a commercial lithium foil (battery grade), supplied by Alfa Aesar. The lithium foil was stored in an Ar-filled glovebox (H_2_O and O_2_ ≤ 0.1 ppm). It was mounted on the ultrahigh vacuum (UHV) sample holder inside the glovebox and transferred to the UHV load lock under an inert atmosphere, avoiding any air exposure. Once in UHV, the lithium surface was scratched with a scalpel connected to a wobble stick, at a base pressure in the 10^–9^ Torr range. The lithium was then transferred to the analysis chamber, and its cleanliness was verified by XPS.

Gas dosing was performed using a precise leak valve. Before dosing, the line was baked to a temperature above 120 °C for at least 24 h, and it was purged three times with a Drytel 1025 pump until the pressure was no longer decreasing, stabilizing at pressures in the 10^–6^ Torr range, before refilling. N_2_ gas (5.0 research purity from Praxair, equivalent to 99.999% purity) was dosed at five different pressures: 0.1, 10, 100, 400, and 800 mTorr, equivalent approximately to 1.3 × 10^–4^, 1.3 × 10^–2^, 1.3 × 10^–1^, 5.3 × 10^–1^, and 1.1 mbar, respectively. Each dosing was performed on a UHV cleaned Li foil, and gas exposure lasted for approximately 50 min. To further study the surface evolution, nitrogen gas was codosed with O_2_ gas (5.0 research purity from Praxair, equivalent to 99.999% purity), CO_2_ gas (5.0 research purity from Praxair, equivalent to 99.999% purity), and H_2_O vapor (Millipore water, 18.2 MΩ). CO_2_ and O_2_ gas lines were cleaned in the same way as for N_2_ gas line. For H_2_O vapor dosing, Millipore H_2_O was poured into a quartz bulb connected to the main chamber through a leak valve. Before dosing, three freeze–pump cycles were performed to degas the water: it was frozen using liquid nitrogen, the residual gases were removed with the Drytel 1025 pump, and the water was allowed to return to liquid before repeating the cycle. The codosing was carried out following a two-stage procedure, where nitrogen gas was introduced to the chamber at a pressure of 400 mTorr after leaking 0.1 mTorr of the codosing gas. Gas dosing experiments were performed at room temperature. To reproduce these experiments reliably, the same pressure, purity, temperature, and exposure time should be maintained to ensure comparable results. XPS high-resolution scans under UHV conditions of O 1s, N 1s, C 1s, and Li 1s regions were collected for the clean and the dosed surfaces. During dosing, the surface composition was determined using 600 eV photons while following N 1s, O 1s, and C 1s XPS spectra evolution, except for 0.1 mTorr N_2_ and 10 mTorr N_2_ doses, where C 1s evolution was not collected. Spectra were analyzed with the CasaXPS version 2.3.19PR1.0 (Casa Software Ltd., Teignmouth, UK). Based on reported values, binding energy calibration was made with the position of Li^0^ binding energy in the Li 1s spectrum at 55.0 eV, lithium carbonate binding energy in the C 1s spectrum at 292.6 eV, and LiOH binding energy in the O 1s spectrum at 533.8 eV.^18,20^ N 1s experimental data were fitted using GL(30) Voigt profiles (30% Lorentzian and 70% Gaussian convolution), where fwhm was constrained to be the same for all the components forming it, and binding energy uncertainties were set to ± 0.1 eV. For the background, Shirley function was used.

## Results

The evolution of nitrogen species on the Li surface during the nitridation experiments at 5 different pressures of N_2_ is depicted in Figure 1. Before each nitridation, Li surface was cleaned under UHV conditions as described in the experimental section, ensuring that each nitrogen dose experiment started with metallic lithium on the surface. Lithium shows no interaction with nitrogen at the lowest gas pressure. At 10 mTorr N_2_ gas, a slight increase in the photoelectron intensity (close to background levels) of N 1s is observed at binding energies above 400 eV, which is higher than that corresponding to Li_3_N.^18^ At 100, 400, and 800 mTorr N_2_ gas, four distinct photoelectron peaks emerged in the N 1s spectra at the following binding energies: 395.3 ± 0.1, 397.5 ± 0.1, 400.0 ± 0.1, and 401.6 ± 0.1 eV. The peak at 395.3 ± 0.1 eV corresponds to Li_3_N,^18^ and assignment of the other three peaks is detailed below in the discussion section. Regarding the N_2_ gas phase photoelectron peak, it is shown in the spectra represented in Figure S1, where it is clearly identified for pressures ≥10 mTorr.

**Figure 1:**
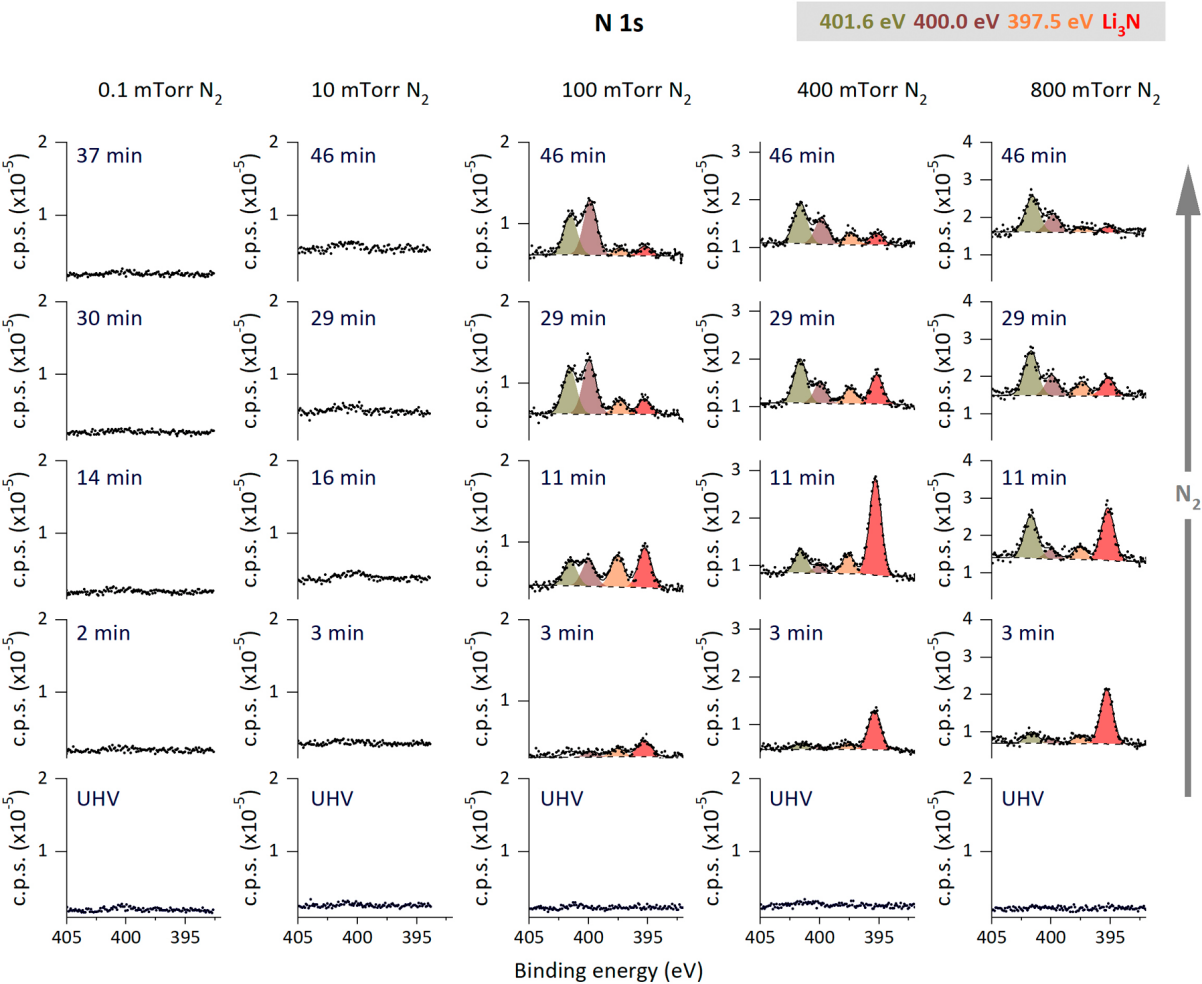
Evolution of N 1s XPS spectra of a metallic lithium surface collected while dosing N_2_ gas at different pressures of 0.1, 10, 100, 400, and 800 mTorr. Data were collected at a photon energy of 600 eV. In the spectra, the fitted curve (line) follows experimental data (dots), and the background signal is represented by a dashed line.

The time evolution of the normalized photoelectron yield area of the four peaks in the N 1s spectra detected during nitridation at 100, 400, and 800 mTorr is represented in Figure 2a. At these three pressures, the photoelectron yield area corresponding to Li_3_N reaches a maximum after 10 min of N_2_ dosing, and then starts to deplete; after an additional 30–40 min of reaction, the nitrogen spectrum is dominated by the components with binding energies of 400.0 and 401.6 eV. Nondestructive depth profiling photoemission measurements, represented in Figure 2b, show the N 1s spectra of Li foil after gas treatment at these three N_2_ gas pressures, which were collected under UHV conditions at three depths, i.e., at three different photon energies. For all the samples studied, the higher the photon energy is, the lower the photoelectron yield intensity associated with the peak at 401.6 eV. This implies that the nitrogen compound at 401.6 eV is in the outermost surface layer, and this behavior is reproduced for the three highest pressures studied.

**Figure 2:**
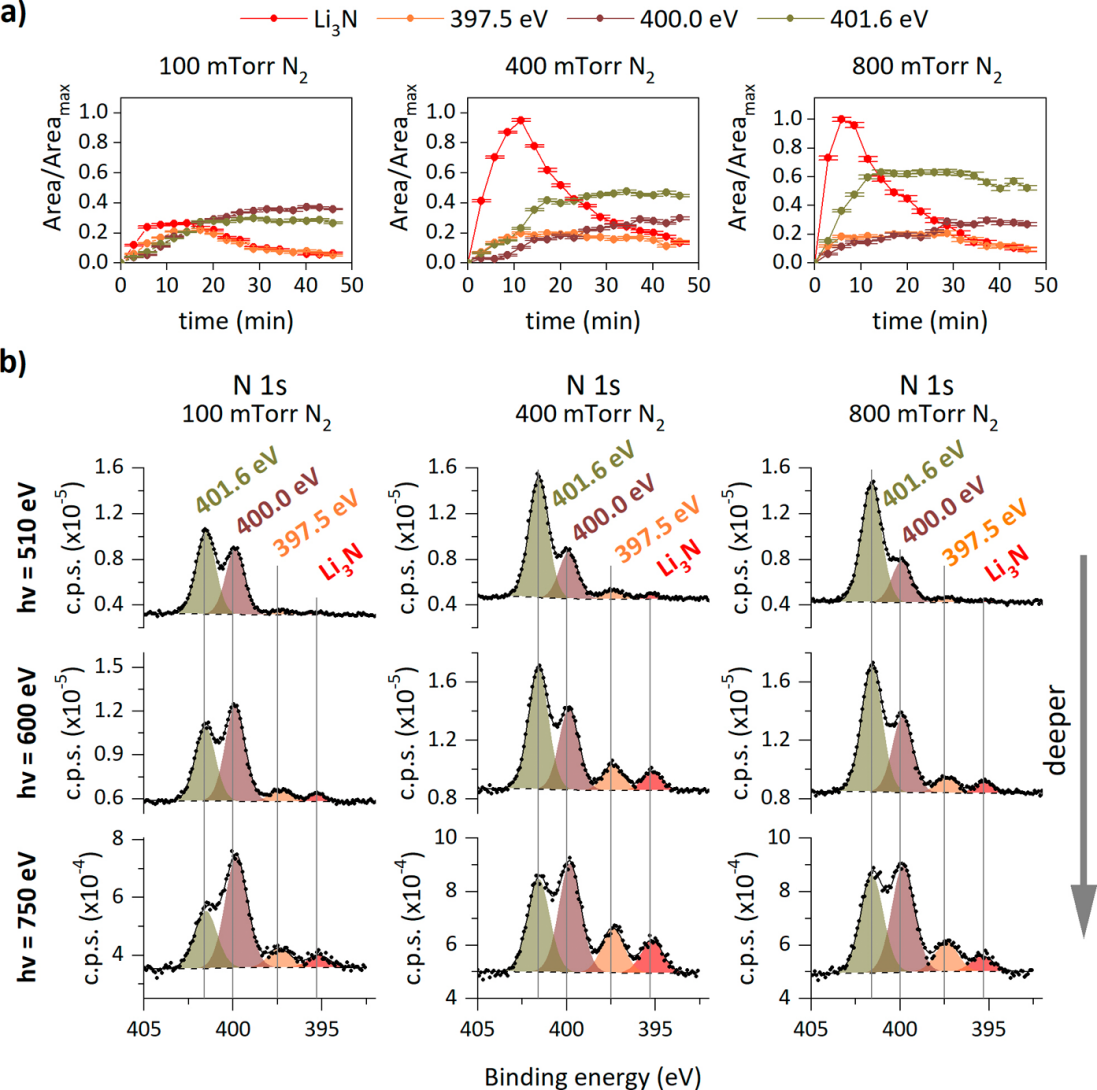
(a) Evolution of the area-normalized relative yield of N 1s XPS components of the metallic lithium surface measured while dosing N_2_ gas at 100, 400, and 800 mTorr with a photon energy of 600 eV. The areas are corrected to exclude the attenuation produced by N_2_ gas on the photoelectron yield. The maximum area used for normalization (*A*_max_) corresponds to a Li_3_N area at an 800 mTorr N_2_ gas dose near minute six. (b) XPS N 1s spectra showing depth profiling of the metallic lithium treated at three different pressures of N_2_ gas for approximately 50 min and measured under UHV conditions. In the spectra, the fitted curve (black line) follows experimental data (dots), and the background signal is represented by a dashed line.

Variation in the surface chemical composition after the reaction can be studied by comparing the Li 1s, C 1s, O 1s, and N 1s core levels before and after nitridation, as shown in Figure 3. The metallic nature of the clean surfaces can be proved by the plasmon loss structures observed in the Li 1s spectra around 63 eV.^18^ The probing depth on all the spectra corresponds to ca. the first 3 nm of the lithium surface, as the spectra were obtained by adjusting the photon energy to maintain a similar kinetic energy of the collected electrons for each region. The estimated surface coverage for all clean surfaces, assuming a bilayer Li_2_O/Li surface and calculated as described in a previous work from the Li 1s spectra measured at 280 eV,^20^ is 4 ± 1 Å Li_2_O. The high surface sensitivity of our measurement is due to the low photon energy used. For example, in the case of a cleaned sample, if measured at a photon energy of 750 eV instead of 280 eV, the oxidized contribution in the Li 1s spectrum would not be as clearly detected, which can be seen in the spectra represented in Figure S2. In Figure 3, the signal corresponding to N-based species is very weak in the spectra collected at the two lowest pressures, and only at pressures above 10 mTorr N_2_ is Li_3_N detected. However, the C 1s and O 1s spectra indicate the formation of oxygen- and carbon-based species. In Figure 3, for pressures ≥10 mTorr, and based on reported binding energies,^18^ the O 1s spectra indicate that Li_2_O grows and that a new peak emerges with a main binding energy that aligns with that of Li_2_O_2_ and LiOH. In the C 1s spectra, the formation of Li_2_CO_3_ is observed.^20^ Additionally, the Li main peak binding energy shifts toward high binding energy after nitrogen exposures of 10, 100, 400, and 800 mTorr, indicating that Li_3_N is not dominant on the surface, as its binding energy is lower than that of Li^0^.^18^ Using the photoelectron areas from the spectra in Figure 3, we calculated the atomic concentration for both clean and dosed surfaces (calculation details can be found in the SI), and we also estimated the detection limit of each element for our experimental conditions (details on the limit of detection calculation can be found on the SI), which are 0.13 ± 0.01% for oxygen, 0.19 ± 0.01% for carbon, and 0.18 ± 0.04% for nitrogen. Table S2 presents the concentration for oxygen, carbon, lithium, and nitrogen, revealing that the surface is primarily composed of Li- and O-based species, likely corresponding to Li_2_O, LiOH, and/or Li_2_O_2_. Notably, the nitrogen concentration in the surface remains below 2% in all cases. In fact, at 10 mTorr the nitrogen concentration is within the detection limit range. Thus, during nitridation, the lithium surface is mainly oxidized by gases other than nitrogen.

**Figure 3:**
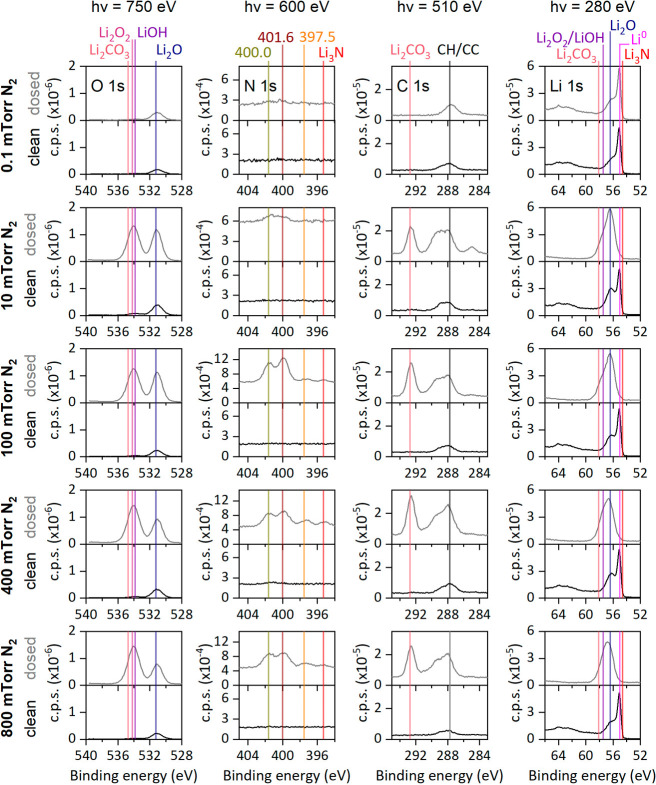
O 1s, N 1s, C 1s, and Li 1s XPS spectra of the Li foil (clean) and the foil after exposure to N_2_ gas at 0.1, 10, 100, 400, and 800 mTorr N_2_ for approximately 50 min. The photoelectrons of the four core levels from the figures have similar kinetic energy (ca. 220 eV), to ensure the collected photoelectrons are coming from the same depth, which in this case corresponds to the first ca. 3 nm of the surface. Spectra were measured under UHV conditions. Binding energies of indicated compounds are taken from literature.^18,20^

Mass spectrometry data collected during nitridation were evaluated to determine why lithium was oxidized by non-N_2_ gases. The mass spectroscopy data, represented in Figure S3, show that when dosing 99.999% pure N_2_ gas into the reaction chamber, CO_2_, H_2_O, and O_2_ are present as impurities. These three gases are known to be reactive toward lithium.^21–23^ Considering the purity of the gases used in this study, one can estimate that the CO_2_, O_2_, and H_2_O impurities are 5 orders of magnitude lower than N_2_ gas. Here, we should mention that this estimate assumes no additional impurities were introduced through the gas lines. We cannot directly verify this, because the RGA is located in the second differential pumping stage, and its mass spectrometry data cannot be directly converted into absolute partial pressures without correcting for factors such as ionization cross sections. However, all gas lines were purged to vacuum levels in the 10^–6^ Torr range and baked before their use, as detailed in the experimental section, to reduce the contamination level in the lines to a lower order than that of the source gas. For this reason, we consider that the impurity level in the reaction chamber should be approximately that of the gas bottle.

To understand the influence of the starting lithium surface composition on the nitridation reaction, we modified (oxidized) the surface of the lithium by dosing O_2_, CO_2_, and H_2_O gases at 0.1 mTorr before introducing N_2_ to the chamber at 400 mTorr. Each two-dose stage experiment started with a cleaned Li surface. The evolution of the N 1s photoelectron line for each set of conditions is shown in Figure 4, together with the O 1s spectra of preoxidation with O_2_ and H_2_O and the C 1s spectra of preoxidation with CO_2_. Comparison of the XPS spectra corresponding to the clean and dosed surfaces, their atomic concentrations, and the mass spectrometry data collected during the dosing experiments are represented in Figures S4, S5, and Table S3, respectively. All the dosed gases oxidized the lithium surface. Based on reported binding energies,^18,20^ the added gases, and the binding energy positions of the new photoelectron peaks that emerged in the spectra shown in Figure 4, we identified the growth of Li_2_O and Li_2_O_2_ during O_2_ gas preoxidation, Li_2_CO_3_ during CO_2_ gas preoxidation, and LiOH during H_2_O vapor preoxidation. Regarding nitrogen gas, the N 1s spectra from Figure 4 indicate that all the codosed gases almost completely inhibit the interaction of the Li surface with N_2_. In the final surface XPS spectra of the mixed doses (Figure S4), for the surface preoxidized by O_2_ gas, no signal increases in the N 1s spectra were observed. For H_2_O and CO_2_ gases, a broad peak formed at binding energies above 400 eV, which did not correspond with the binding energy associated with Li_3_N. Thus, Li_3_N is not detected in any of the studied cases. This result shows no evidence to support the hypothesis that contaminants (especially water) catalyze the formation of Li_3_N upon exposure of a lithium surface to N_2_ gas, aligning with the findings of previous work.^16^

**Figure 4:**
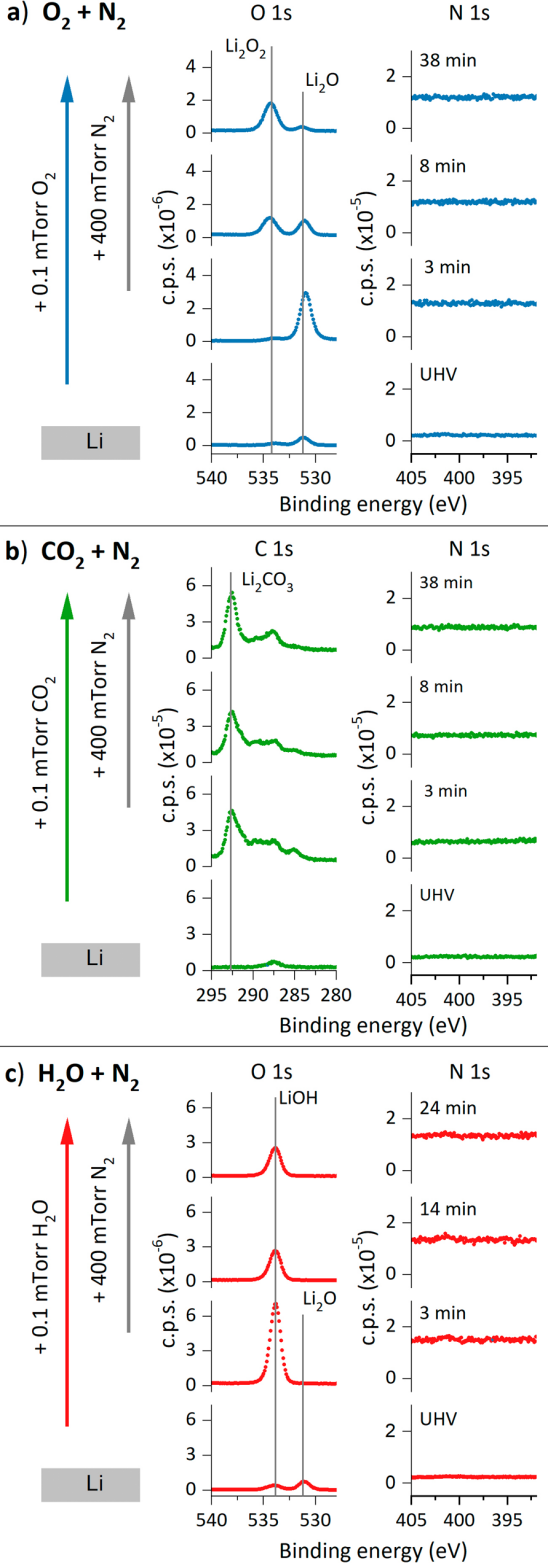
Evolution of XPS spectra of the metallic lithium surface measured during two-stage dosing of (a) O_2_ gas, (b) CO_2_ gas, and (c) H_2_O vapor with N_2_ gas. All data were collected at a photon energy of 600 eV. Binding energies of indicated compounds were obtained from literature refs 18 and 20 .

## Discussion

Thermodynamically, the formation of Li_3_N on the Li surface is feasible, as the free energy of the reaction is negative across all the studied pressures (Table S4). However, Li_3_N was not detected at pressures below 100 mTorr. This implies that to overcome the activation energy of this reaction, a minimum nitrogen gas pressure is required to sufficiently increase the collision probability, which, in addition to the subsequent kinetic energy transfer, facilitates the reaction from the kinetics point of view; in our case, Li_3_N was only detected at pressures of 100 mTorr or higher. This observation clarifies why lithium was not successfully nitridated in some previous studies, as the reactions were conducted with nitrogen gas pressures below 0.1 mTorr.^18,19^ Moreover, from the two-state dosing experiments and previous studies,^21–23^ we observe that nitrogen gas reacts differently with lithium than the other major reactive atmospheric gases: O_2_, CO_2_, and H_2_O all react at significantly lower pressures than those required for nitrogen. These results also highlight the importance of performing experiments under relevant pressure conditions to better address the reactivity of a surface toward a gas.

In this nitridation study, we hypothesized that a layer of Li_3_N would form on the surface of lithium. However, surprisingly, except in the 0.1 mTorr N_2_ dose experiment, where the lithium remained metallic, the final surface was predominantly composed of lithium and oxygen, with an atomic nitrogen concentration below 2% (Table S2). The absence of surface dominated by Li_3_N was also confirmed by evaluating the main Li 1s binding energy peak after nitridation, as shown in Figure 3. The peak position aligns with Li_2_O for the reactions at 10 mTorr and 100 mTorr N_2_ gas, and it shifts to a position between Li_2_O and LiOH/Li_2_O_2_ for those at 400 mTorr and 800 mTorr N_2_ gas. Besides, the disappearance of the plasmon-loss feature further supports that after nitridations at pressures ≥10 mTorr N_2_ there is an increased emergence of surface contaminants, and that the lithium on the probing volume has been oxidized. Additionally, the lower carbon concentration observed in the surface analysis of dosed lithium compared to oxygen (Table S2), together with lithium’s known high reactivity with O_2_ and H_2_O,^21,23^ indicates that lithium preferentially reacts with these species despite having nitrogen pressures approximately five orders higher than those of O_2_ and H_2_O in the chamber.

Another notable observation concerns the nitrogen-based species formed on the lithium surface. During lithium nitridation, instead of only producing Li_3_N, at least three additional species were detected in the N 1s XPS spectra at 397.5 ± 0.1, 400 ± 0.1, and 401.6 ± 0.1 eV (Figure 1). The binding energies (400 ± 0.1 and 401.6 ± 0.1 eV) of two of those species are in the range reported for graphitic carbon nitrides or nitrogen-doped carbons.^24–26^ Previous reports claim that exothermic reactions occur between Li_3_N and CO_2_ gas,^27^ leading to the production of species such as lithium cyanamide (Li_2_CN_2_), graphitic carbon nitride (g-C_3_N_4_), and amorphous C_*x*_N_*y*_.^27,28^ Based on this information, and considering the decrease in the Li_3_N signal (Figure 2a) in addition to the detection of CO_2_ gas via mass spectrometry (Figure S3), we conclude that the formed Li_3_N rapidly reacts with trace CO_2_ gas in the chamber, leading to the formation of new carbon- and nitrogen-based species. To assess whether the X-ray beam influences the reactivity of Li_3_N, we referred to an experiment in which lithium was nitridated in a different UHV setup, with nitridation occurring in a separate chamber from the analysis.^29^ The resulting N 1s spectrum is shown in Figure S6. In that study, photoelectron peaks near 400 and 397.5 eV were also detected, along with the peak corresponding to Li_3_N, indicating that the observed products in our work were not induced by the X-ray beam.

Based on the area evolution over time shown in Figure 2a, the peaks at (401.6 ± 0.1) and (400.0 ± 0.1) eV could be attributed to two nitrogen species from the same compound, as they exhibit the same trend. However, depth profiling experiments, represented in Figure 2b, reveal an inversion in the area ratio of these two nitrogen species closer to the surface. This suggests that these two nitrogen species are independent and do not originate from the same compound, as their area ratio would otherwise remain constant with decreasing depth. Considering reported binding energies, we associate the peak at 401.6 eV with quaternary nitrogen and the peak at 400.0 eV with pyrrolic nitrogen.^24,25^ This last assignment may imply that LiOH also participates in the reaction alongside Li_3_N and CO_2_, as alkaline hydroxides have previously been used to promote pyrrolic nitrogen from nitrogen-doped carbon materials.^30^ Regarding the peak at (397.5 ± 0.1) eV, its area evolution with time (Figure 2a) is independent of the other nitrogen species, suggesting that this peak represents a distinct type of nitrogen. Its binding energy lies outside those reported for nitrogen-doped carbons.^24^ Based on its likelihood of forming,^27^ and its binding energy, similar to that found for the C = N bond in indium cyanamide,^31^ we tentatively associate it with Li_2_CN_2_. Furthermore, since Li_2_CN_2_ acts as a precursor to form carbon nitrides,^32^ this may explain the observed area decrease shown in Figure 2a. However, clear and definitive chemical assignment of the nitrogen-based compounds requires further experimental investigation with additional surface-sensitive techniques. The main observations thus far (namely, the lack of reaction with N_2_ at the lowest pressure studied, the reaction of Li_3_N forming other products, and the surface being dominated by oxygen) are schematically illustrated in Figure 5.

**Figure 5:**
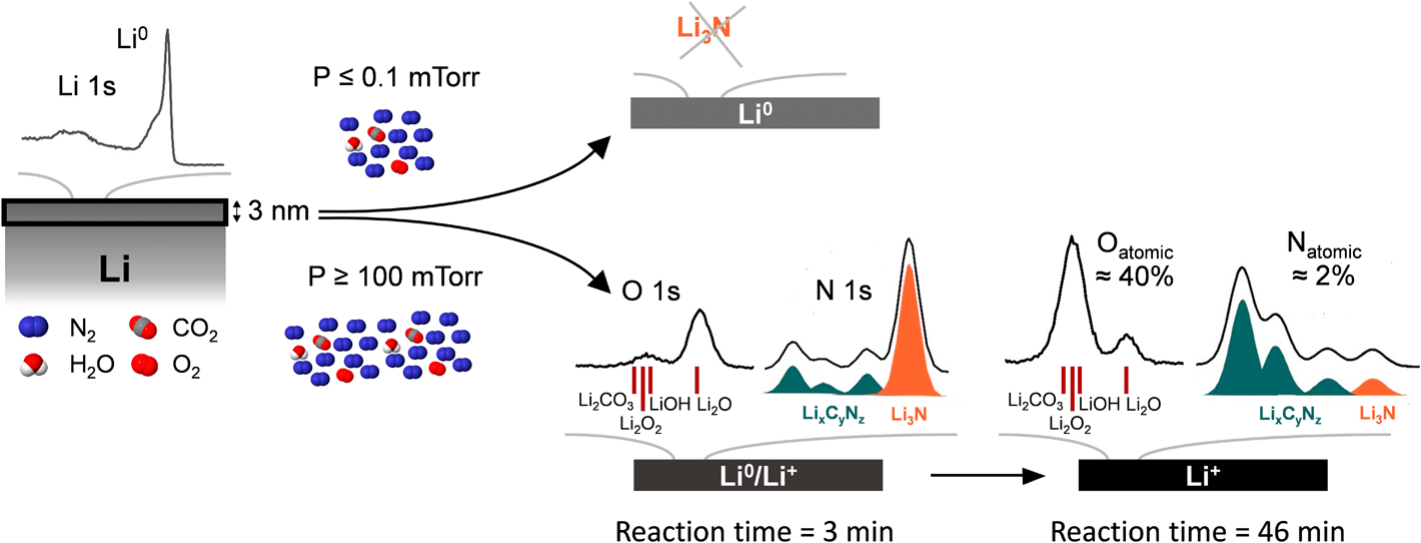
Schematic showing the conditions at which Li_3_N is created on the outermost surface of pure metallic lithium when it reacts with N_2_ gas that contains minor O_2_, CO_2,_ and H_2_O traces (approximately 5 orders of magnitude lower than nitrogen). Li_3_N is only detected when the pressure of N_2_ is ≥100 mTorr and converts to other products that appear consistent with carbon nitrides. In the figure, the N 1s and O 1s spectra correspond to an 800 mTorr N_2_ dose, with the O 1s shown as the raw data and N 1s as the fitted envelope, both measured at a photon energy of 600 eV. The Li 1s spectrum represents the cleaned Li foil, measured at a photon energy of 280 eV.

Interest in carbon nitrides has gained traction in recent years due to their catalytic properties in CO_2_ conversion to chemicals and fuels.^32,33^ Most routes to obtain carbon nitrides require a high-temperature procedure.^33,34^ In our study, carbon nitrides could be formed at room temperature. However, pure lithium nitridation in a CO_2_ atmosphere does not appear to be practical for synthesizing carbon nitrides at room temperature, as they were generated in low quantities in this study. While the studied reaction does not yet produce useable quantities of Li_3_N, it highlights that if high-purity Li_3_N could be generated, the approach could potentially be explored for the synthesis of carbon nitrides as well as for CO_2_ reduction. In carbon nitride synthesis, the role of the N_2_ pressure could be further explored, as our results show it influences the final surface composition: the higher the pressure was, the higher the ratio of the species identified as quaternary nitrogen (binding energy: 401.6 eV) to that identified as pyrrolic nitrogen (binding energy: 400.0 eV).

Regarding the N_2_ gas phase photoelectron peak shown in Figure S1, its binding energy increases from 407.5 ± 0.1 to 408.2 ± 0.1 eV while dosing nitrogen, as indicated in the figure for pressures >10 mTorr. These changes can be explained by modifications of the surface work function, where an increase in the gas-phase binding energy has been correlated with a decrease in the work function,^35^ as expected for Li surface oxidation, which has been reported to lower the work function.^19^

The Li foil used in this study is polycrystalline. We acknowledge that different surface orientation and sites may exhibit varying reactivity, as observed experimentally for other gas/metal reactions and predicted for N_2_/Li.^36,37^ However, this aspect was not addressed in the present work. Lithium is a very soft metal where metastable structures close in energy exist,^17^ and is highly reactive under ambient conditions, making high-quality single crystals with polished surfaces difficult to obtain commercially and challenging to clean under UHV conditions. For this reason, we conducted our experiments on lithium foil with undefined grain size and preferential orientation. A systematic study using well-defined single crystals with specific surface orientations and controlled roughness parameters would be an interesting direction for future work.

As previously mentioned, several experimental works observed that Li_3_N is created on Li foil at room temperature by exposing the foil to N_2_ gas.^5,8^ It is likely that the surface of the lithium used in these other works is oxidized, as the starting lithium surface purity is not mentioned, and it is well-known that Li foil has a native oxidized surface layer even in an inert glovebox environment.^38^ Thus, no metallic lithium is expected to be on the surface, but Li_3_N is formed. This observation seems to be in contradiction to our results. However, an explanation for this could be that nitrogen passes through the oxidized layer of lithium and reacts with Li^0^ in the bulk layer, and the technique applied in this study is not sensitive to it. An indication of this phenomenon was observed by Wu et al.,^5^ as they could only measure surface Li_3_N after removing 13 nm of the foil surface. Ma et al. utilized X-ray diffraction (XRD) to analyze Li_3_N,^8^ which is a bulk-sensitive technique that does not probe the outermost surface of the Li foil. Another point to consider is the porosity of the oxidized layer, which could affect the ability of N_2_ gas to permeate the layer to reach metallic lithium. The hypothesis that H_2_O catalyzes lithium nitridation could be supported by the higher porosity of LiOH compared to, for instance, Li_2_O. This greater porosity could allow more N_2_ to access lithium, accelerating N_2_ consumption. This aligns with our results, as we observed no formation of Li_3_N on the surface when it was covered by LiOH, indicating that LiOH and water do not act as a catalyst but could instead reduce the transport limitations of N_2_ gas. Taking all this into account, we must acknowledge that in our experiments, there is the possibility that Li_3_N formed beneath the oxidized layer at a depth that we were unable to probe with the applied technique, as our surface sensitivity was limited to 3 nm. Theoretical insight into the diffusion process of N_2_ in this system would be very useful in guiding the design of new experiments that probe beyond the subsurface region. Notably, for the codose experiments, final N 1s spectra (Figure S4) show that some nitrous species were formed for the CO_2_ and H_2_O preoxidized Li foils. If we consider that those species are created from the interaction between Li_3_N and CO_2_ gas, it would indicate that Li_3_N remained undetected due to its depth below the surface and/or being consumed prior to detection. Further experiments employing higher nitrogen pressures and utilizing both surface- and bulk-sensitive analytical techniques are needed to further determine optimal conditions for lithium nitridation. In addition, while multiscale modeling with 5-order of magnitude difference in reactant concentration is challenging and unconstrained ab initio molecular dynamics applied to metal surfaces can only reveal reaction mechanisms with low reaction barriers,^39^ it is suggested to focus on modeling multiple reaction steps and multilayer surface films that drive the electric double layer,^40^ crucial for the understanding of ionic transport and reactivity in electrochemical reactions. At the same time, calculating the electronic voltage, which is a measure of the Fermi energy or work function, remains crucial for any DFT determination.^19,39^ Initial work demonstrating a digital twin for chemical science surfaces also lays the foundation for future work capable of incorporating surface reconstruction and transformations for different reactions and surface chemical reactions.^41^ The experiments conducted in this study were crucial in revealing the chemistry of lithium following nitridation with minimal gas impurities. This study extends previous efforts to identify specific surface compositions that could improve the performance of metallic lithium anodes, laying the groundwork for future studies involving electrochemical tests on modified lithium anodes with well-characterized surfaces in highly controlled environments. Our results also highlight potential challenges with Li–N_2_ battery rechargeability, as the high reactivity of Li_3_N could lead to complications during the charging process.

## Conclusions

In this work, we studied the nitridation of a metallic lithium surface over a pressure range of 0.1–800 mTorr, evaluating its chemical surface composition throughout the reaction using APXPS. Additionally, we monitored the composition of the reactive gases via mass spectrometry. Our analysis clarified several key aspects of the lithium nitridation reaction. First, we found that a minimum N_2_ gas pressure between 0.1 and 10 mTorr is required to detect any nitrogen-related products on the surface. We also observed that even minimal impurities can significantly alter the final surface composition. Due to lithium’s strong reaction affinity to O_2_ and H_2_O, after nitridation with trace amounts (approximately five magnitudes of order lower) of O_2_, H_2_O, and CO_2_ present alongside N_2_, the surface becomes largely covered by oxygen-related species. As a result, nitrogen constitutes less than 2% of the surface composition. Additionally, Li_3_N reacts with trace CO_2_ gas, forming species that appear consistent with carbon nitrides.

By conducting experiments where the initial lithium surface is oxidized by gases other than N_2_, we also determined that Li_3_N only forms on the surface when metallic lithium is directly exposed to N_2_ gas. Here, we found no evidence to suggest that H_2_O catalyzes the nitridation reaction. Instead, we propose that, when pressure is sufficiently high, N_2_ gas can penetrate the oxidized lithium surface to react with metallic lithium in the bulk layer, which may explain why Li_3_N was detected in previous studies and not in ours. In summary, our study demonstrates that the surface chemistry of lithium nitridation is highly complex. Moreover, using this method may not be feasible to form Li_3_N on a lithium surface. This study provides useful insights for accurately correlating the observed properties of a treated surface, such as improved electrochemical stability of lithium anodes, with the corresponding surface compounds.

## Supplementary Material



## Notes

Details on the calculation of the atomic concentration from the XPS data; details on the calculation of the detection limit of C, O, and Li; N 1s XPS spectra during lithium nitridation showing the N_2_ gas photoelectron peak; Li 1s spectra collected at two photon energies; mass spectrometer data during lithium nitridation; XPS spectra of the clean and dosed lithium surface for the codose experiments; mass spectrometer data during the codose experiments; N 1s XPS spectrum of a lithium foil exposed to N_2_ gas; physical parameters to calculate the atomic concentration of the surface; atomic concentration of clean and treated Li foil; and Gibbs free energy formation of Li_3_N (PDF)

## ◆

Department of Polymers and Advanced Materials, University of the Basque Country UPV/EHU, 20018 Donostia/San Sebastián, Spain

## ¶

International Iberian Nanotechnology Laboratory, Av. Mestre José Veiga, s/n, 4715-330 Braga, Portugal.
