# Supplementary material for: Probing the Surface Chemistry of Lithium Nitridation
Source: J Am Chem Soc. 2025 Oct 27;147(44):40398–406. doi: 10.1021/jacs.5c11781 (PMC12593374; doi:10.1021/jacs.5c11781)
Supplement: Supplementary file 1 [file ja5c11781_si_001.pdf]

SUPPORTING INFORMATION

Probing the Surface Chemistry of Lithium Nitridation

Ane Etxebarria,[a,b,c] Pinar Aydogan Gokturk,[c,d] Yifan Ye,[c,e,f] Philip N. Ross,[g] Ethan J. Crumlin,*[c,f] and Miguel Ángel Muñoz-Márquez*[a,h]*

a. Centre for Cooperative Research on Alternative Energies (CIC energiGUNE), Basque Research and Technology Alliance (BRTA), Vitoria-Gasteiz 01510, Spain

b. Departamento de Física de la Materia Condensada, Facultad de Ciencia y Tecnología, Universidad del País Vasco, UPV/EHU, Bilbao 48080, Spain

c. Advanced Light Source, Lawrence Berkeley National Laboratory, Berkeley, California 94720, United States

d. Department of Chemistry, Koc University, Istanbul 34450, Turkiye

e. Joint Center for Artificial Photosynthesis, Lawrence Berkeley National Laboratory, Berkeley, California 94720, United States

f. Chemical Sciences Division, Lawrence Berkeley National Laboratory, Berkeley, California 94720, United States

g. Materials Sciences Division, Lawrence Berkeley National Laboratory, Berkeley, California 94720, United States

h. School of Science and Technology, Chemistry division, University of Camerino, Camerino, Macerata 62032, Italy

Atomic concentration from the XPS data

The collected intensity in the analyzer for an element (A) in a homogeneous sample is:

$$I = TD_{eff}(A\cos\theta)\Delta\Omega F\sigma N\lambda \qquad \text{(eq S1)}$$

where T is the analyzer transmission function, D_{eff} is the efficiency of the detector, A is the analyzed area, θ is the emission angle of photoelectrons with respect to the surface normal, $\Delta\Omega$ is the analyzer solid acceptance angle, F is the flux of X-ray photons, N is the atomic density, σ is the photoemission cross section and λ is the photoelectron inelastic mean free path.[1] In this definition photoelectrons elastic scattering events are neglected.

Using the parameters from Table S1, the areas of the core levels of Figures 3 and S4 with Shirley background, and assuming that due to having a very similar kinetic energy, all the electrons are coming from the same depth, the atomic concentration (N) of the elements on the surface have been calculated from equation S1, which is represented in Tables S2 and S3.

To calculate the error on the obtained atomic concentrations, Monte Carlo simulations were performed in each region using CasaXPS software, which estimates the precision error in calculating the area of a peak above the background signal. The standard deviations obtained for each region were then propagated to determine the standard deviation of the atomic % in each case, and are shown with an error bar in Tables S2 and S3.

Detection limit of C, O, and N

The limits of detection for N, O, and C, under our experimental conditions, were estimated by first calculating their detection limit areas using the following equation

$$A_{LOD,i} = k * \sqrt{BN} \quad \text{(eq S2)}$$

where
$A_{LOD,i}$: area of the limit of detection of element i, counts
k: constant to establish an arbitrary confidence level. We have used k = 2, which corresponds to a confidence level of approximately 95%.[2]
B: background intensity, counts
N: number of data channels in the region used to calculate the background intensity

The parameter √BN was determined with the CasaXPS software from the O 1s collected at 750 eV, C 1s spectra collected at 510 eV, and N 1s spectra collected at 600 eV, of the clean Li surfaces. Then, the detection limit of C, N, and O, in a Li matrix, was calculated with the following equation:[2]

$$x_{LOD_i} = \frac{A_{LOD,i}/s_i}{A_{LOD,i}/s_i + \Sigma(A_j/s_j)} \quad \text{(eq S3)}$$

where
$x_{LOD,i}$: limit of detection of element i, atomic%
s_i: sensitivity factor of element i
A_j: area of element j, counts
s_j: sensitivity factor of element j

To determine the detection limit of oxygen (i = O), a Li matrix was considered, with j corresponding to Li and its area taken from the Li 1s spectra collected at 280 eV for the clean surface. For nitrogen and carbon (i = N, C), a Li + O matrix was considered, since the initial sample surface already contained more than 10% oxygen. In this case, j will be Li and O, with the areas taken from the Li 1s spectra at 280 eV and the O 1s spectra at 750 eV, both measured on clean surfaces.

Regarding the sensitivity factors of each element, they correspond to the product of the cross section and photon flux, which are detailed in Table S1. The indicated errors for each detection limit correspond to the standard deviation across the clean surfaces.

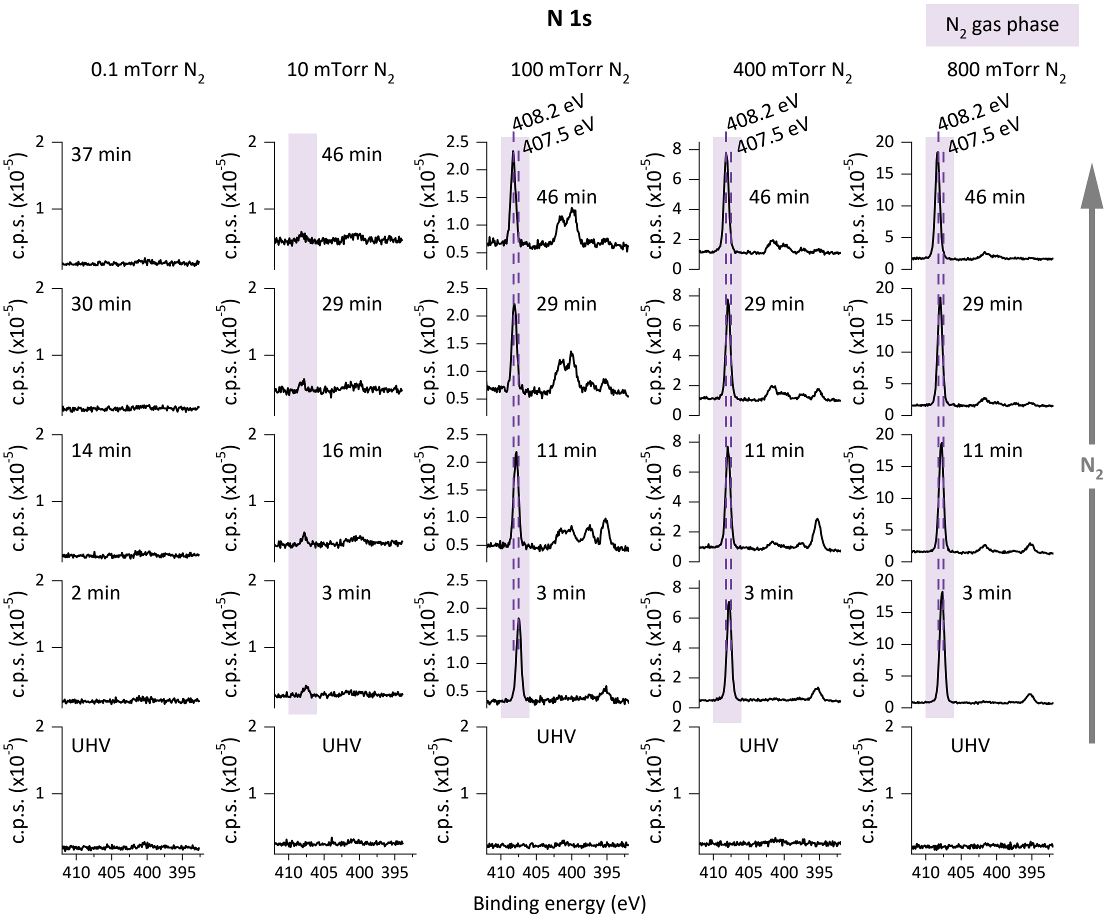

Figure S1. N 1s XPS spectra of a metallic lithium surface collected while dosing N_2 gas at different pressures of 0.1, 10, 100, 400, and 800 mTorr. The area of the spectra where the photoelectron peak of N_2 gas appears is highlighted in light purple.

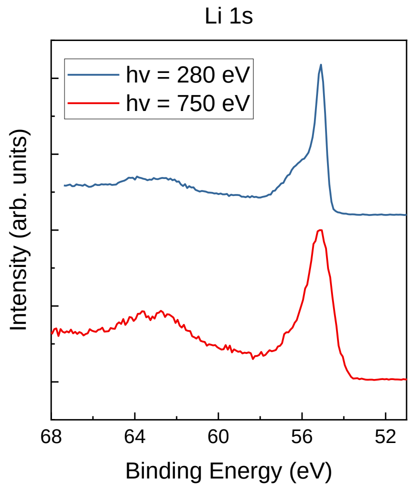

Figure S2. Li 1s XPS spectra of a cleaned Li surface (the same surface used for the 0.1 mTorr N_2 dose experiments) measured at two photon energies: 280 eV (blue) and 750 eV (red).

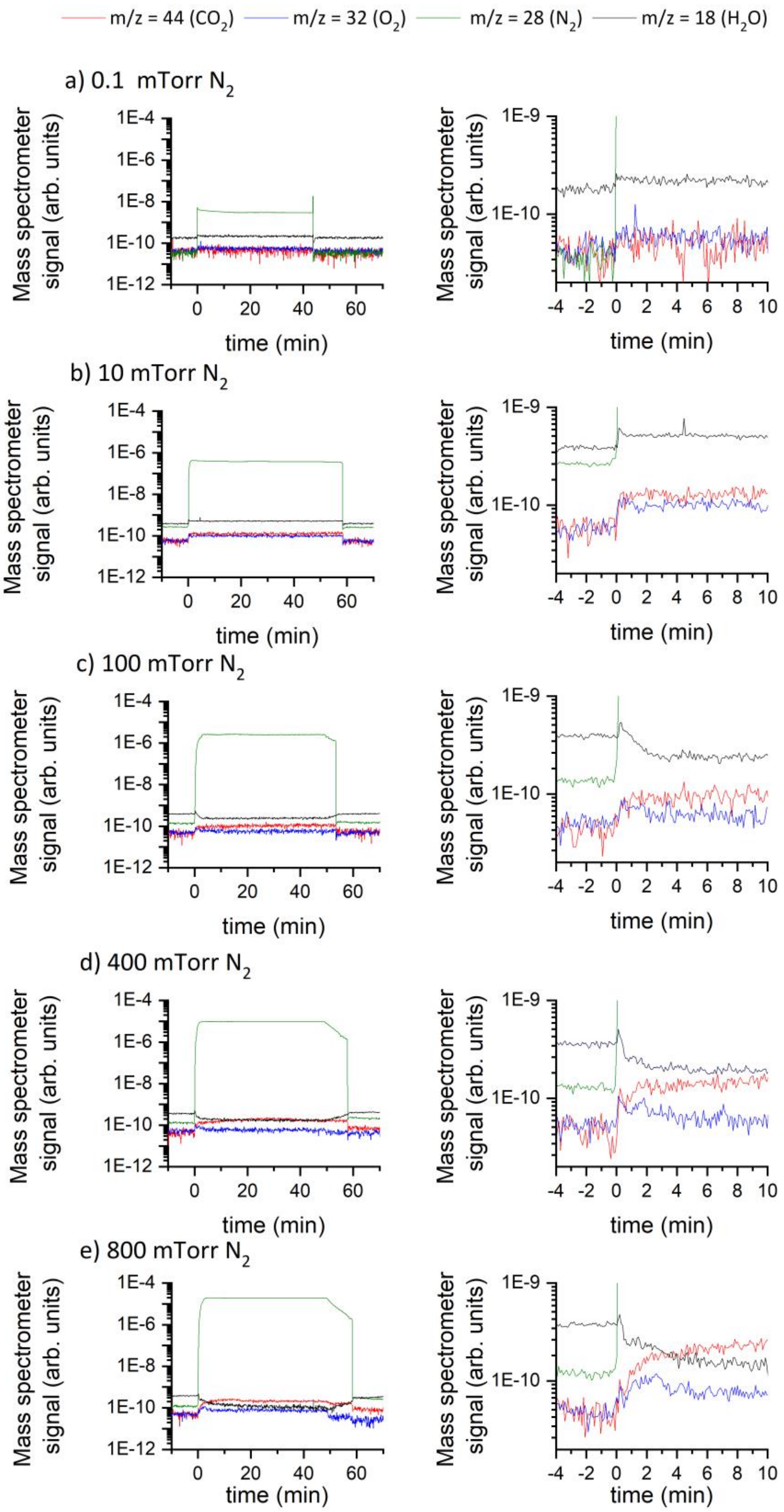
m/z = 44 (CO2)
m/z = 32 (O2)
m/z = 28 (N2)
m/z = 18 (H2O)
a) 0.1 mTorr N2
b) 10 mTorr N2
c) 100 mTorr N2
d) 400 mTorr N2
e) 800 mTorr N2
Mass spectrometer signal (arb. units)
time (min)
1E-4
1E-6
1E-8
1E-10
1E-12
1E-9
0
20
40
60
-4
-2
2
4
6
8
10

Figure S3. Evolution of the partial pressure of the gases inside the APXPS chamber as determined by mass spectrometry when dosing a) 0.1 mTorr N_2, b) 10 mTorr N_2, c) 100 mTorr N_2, d) 400 mTorr N_2, and e) 800 mTorr N_2 for approximately 50 minutes. The partial pressure of each gas is represented with a different color in logarithmic scale, namely green (N_2), red (CO_2), blue (O_2), and black (H_2O). Plots on the right side correspond to a magnification of the evolution of the gases at the beginning of the reaction.

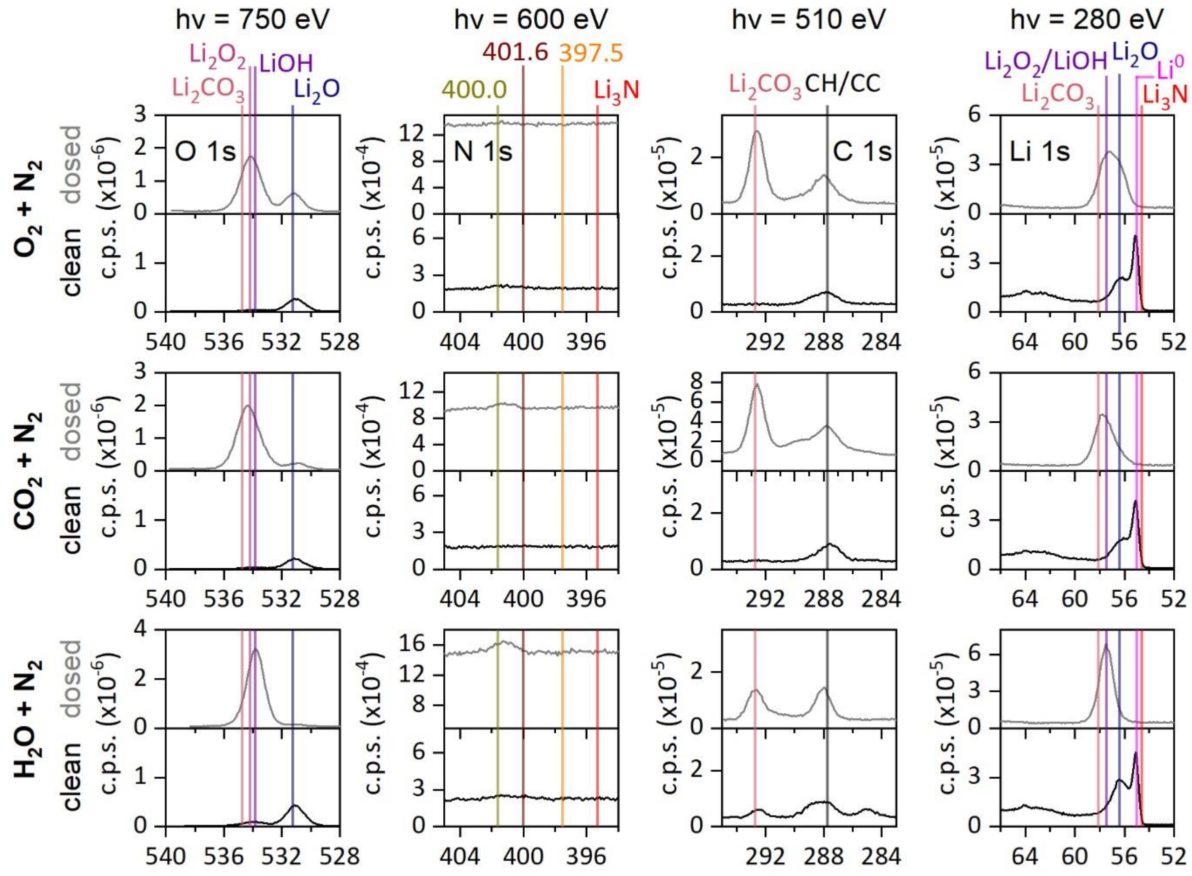

Figure S4. O 1s, N 1s, C 1s, and Li 1s XPS spectra of the Li foil (clean) and the foil after exposure to 0.1 mTorr O_2 and 400 mTorr N_2, 0.1 mTorr CO_2 and 400 mTorr N_2, and 0.1 mTorr H_2O and 400 mTorr N_2 (dosed). The photoelectrons of the four core levels from the figures have similar kinetic energy (ca. 220 eV), to ensure the collected photoelectrons are coming from the same depth, which in this case corresponds to the first ca. 3 nm of the surface. Spectra were measured under UHV conditions. Binding energies of indicated compounds are taken from literature.[3,4]

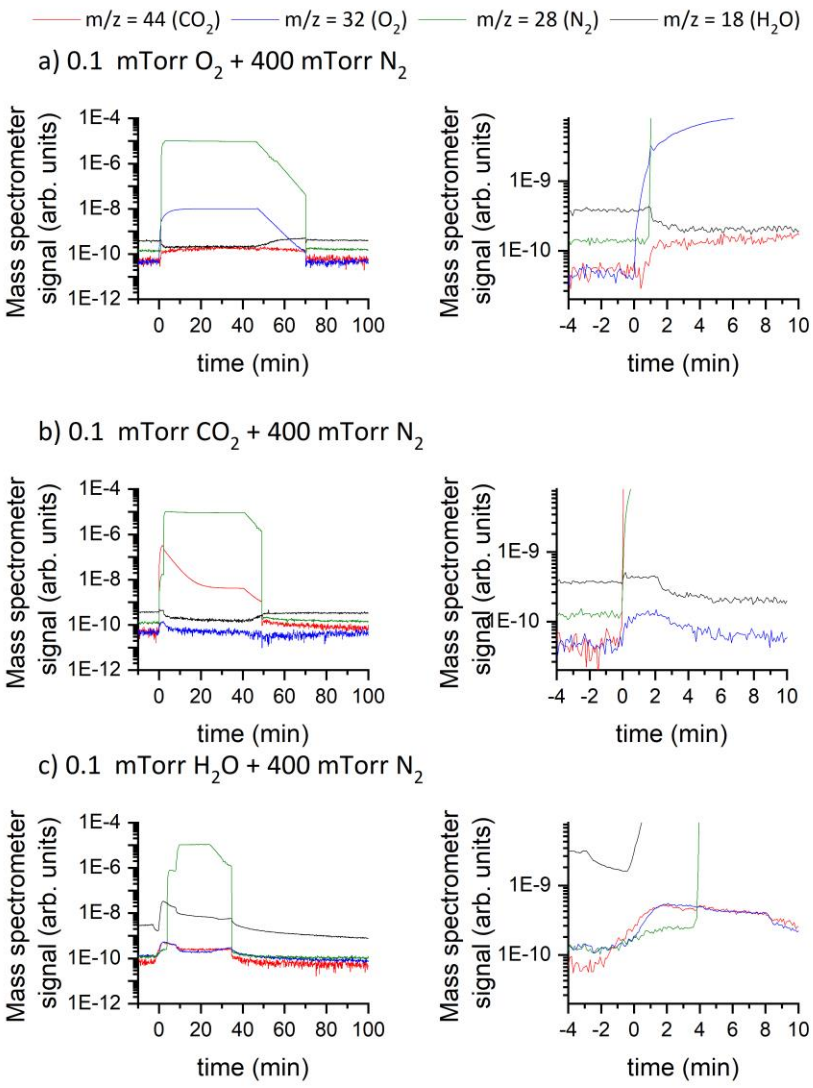

Figure S5. Evolution of the partial pressure of the gases inside the APXPS chamber as determined by mass spectrometry when dosing at two stages a) 0.1 mTorr O_2 and 400 mTorr N_2, b) 0.1 mTorr CO_2 and 400 mTorr N_2, and c) 0.1 mTorr H_2O and 400 mTorr N_2. Plots on the right side correspond to a magnification of the evolution of the gases at the beginning of the reaction.

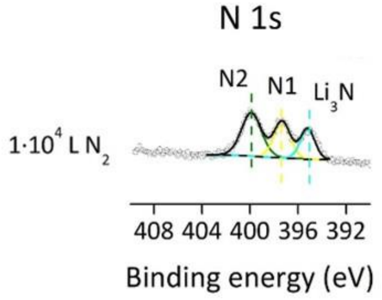

Figure S6. N 1s XPS spectrum (measured with a non-monochromatic Mg Kα photon source) of a UHV-cleaned lithium foil after a 10^4 Langmuir N_2 dose. Nitridation was done in a preparation chamber, separated from the analysis chamber where XPS was measured. In the spectra, the fitted curve (black line) follows experimental data (dots), and the background is represented by a dashed line. Figure modified from ref [5]. Available under a CC-BY 3.0 license. Copyright 2020 Ane Etxebarria.

Table S1. Physical parameters used to calculate the atomic concentration of the surface layers.

Photon energy (eV)	Core level	Cross section[I] (Mbarn)	Photon Flux[II]
750	O 1s	0.2931	0.470
600	N 1s	0.2763	0.720
510	C 1s	0.2563	0.705
280	Li 1s	0.1103	0.231

[I] from database[6]

[II] normalized value of photon flux (photons s^{-1} mA^{-1} μm^{-1}), experimental parameter measured in beamline 9.3.2 of Advanced Light Source.

Table S2. Atomic concentration of clean and nitrogen dosed Li surface, which represents the atomic composition of the first ca. 3 nm of the surface. Data has been calculated from the area of the photoelectron peaks in Figure 3 and following equation S1.

**N_2 pressure (mTorr)**	**Atomic %**							
	**Li**		**O**		**C**		**N**	
	Clean	Dosed	Clean	Dosed	Clean	Dosed	Clean	Dosed
0.1	86.89 ± 0.03	83.64 ± 0.03	10.30 ± 0.02	13.45 ± 0.02	2.81 ± 0.02	2.92 ± 0.02	0.00	0.00
10	80.76 ± 0.03	55.97 ± 0.03	16.91 ± 0.02	38.56 ± 0.02	2.33 ± 0.02	5.33 ± 0.01	0.00	0.14 ± 0.01
100	86.15 ± 0.03	55.47 ± 0.03	11.78 ± 0.02	38.26 ± 0.02	2.08 ± 0.02	5.03 ± 0.01	0.00	1.23 ± 0.01
400	82.93 ± 0.03	53.65 ± 0.03	14.27 ± 0.02	38.34 ± 0.03	2.80 ± 0.02	6.39 ± 0.02	0.00	1.61 ± 0.01
800	85.96 ± 0.03	54.01 ± 0.03	12.29 ± 0.02	38.96 ± 0.03	1.76 ± 0.02	5.35 ± 0.01	0.00	1.68 ± 0.02

Table S3. Atomic concentration of clean and dosed Li surface in the codose experiments, where 0.1mTorr codosing gas was added before 400 mTorr N_2. The table represents the atomic composition of the first ca. 3 nm of the surface. Data has been calculated from the area of the photoelectron peaks from Figure S4 and following equation S1.

**Codosed gas**	**Atomic %**							
	**Li**		**O**		**C**		**N**	
	Clean	Dosed	Clean	Dosed	Clean	Dosed	Clean	Dosed
O_2	82.91 ± 0.04	50.06 ± 0.04	14.34 ± 0.03	44.94 ± 0.04	2.58 ± 0.03	5.00 ± 0.01	0.17 ± 0.01	0.00
CO_2	83.95 ± 0.04	37.65 ± 0.04	12.83 ± 0.03	46.50 ± 0.03	3.22 ± 0.03	15.72 ± 0.02	0.00	0.13 ± 0.01
H_2O	77.52 ± 0.03	47.15 ± 0.05	18.59 ± 0.03	50.14 ± 0.05	3.74 ± 0.03	2.48 ± 0.01	0.15 ± 0.01	0.23 ± 0.01

Table S4. Gibbs free energy of the formation of Li_3N at several nitrogen pressures. The data has been calculated from thermodynamic data available in reference.[7]

**Reaction**	**N_2 pressure (mTorr)**	**ΔG (kJ/mol)**
**$3Li + 0.5N_2(g) \rightarrow Li_3N$**	0.1	-109.041
	10	-114.743
	100	-117.594
	400	-119.31
	800	-120.168

REFERENCES

(1) Jablonski, A. Evaluation of Procedures for Overlayer Thickness Determination from XPS Intensities. *Surf Sci* **2019**, *688*, 14–24.

(2) Shard, A. G. Detection Limits in XPS for More than 6000 Binary Systems Using Al and Mg Kα X-Rays. *Surface and Interface Analysis* **2014**, *46* (3), 175–185.

(3) Wood, K. N.; Teeter, G. XPS on Li Battery Related Compounds: Analysis of Inorganic SEI Phases and a Methodology for Charge Correction. *ACS Appl Energy Mater* **2018**, *1*, 4493–4504.

(4) Etxebarria, A.; Yun, D.; Blum, M.; Ye, Y.; Sun, M.; Crumlin, E. J. Revealing in Situ Li Metal Surface Evolution upon Exposure to CO_2 Using Ambient Pressure X-Ray Photoelectron Spectroscopy, *ACS Appl. Mater. Interfaces* **2020**, 12 (23), 26607−26613.

(5) Etxebarria Dueñas, A. Study of Li Metal Anode Surface. Interaction with Atmospheric Gases and Impact of Impurities in Electrochemistry. PhD dissertation, Centre for Cooperative Research on Alternative Energies (CIC energiGUNE) and University of the Basque Country (UPV/EHU), **2020**.

(6) Atomic Calculation of Photoionization Cross-Sections and Asymmetry Parameters. https://vuo.elettra.eu/services/elements/WebElements.html

(7) JANAF Thermochemical Tables, J. Phys. Chem. Ref. DATA, Vol 14, Suppl. 1, 1985.
